# Supplementary material for: The gene “degrees of kevin bacon” (dokb) regulates a social network behaviour in Drosophila melanogaster
Source: Nat Commun. 2024 Apr 30;15:3339. doi: 10.1038/s41467-024-47499-8 (PMC11061139; doi:10.1038/s41467-024-47499-8)
Supplement: Supplementary file 1 — Supplmentary Information [file 41467_2024_47499_MOESM1_ESM.pdf]

The Gene "*degrees of kevin bacon*" (*dokb*) Regulates a Social Network Behaviour in *Drosophila melanogaster*

Supplementary Tables

Supplementary Table 1: Genes expressed in the 474kb locus within the CNS of CS and OR flies, as determined by RNAseq. Genes that are significantly differentially expressed between the two strains are highlighted in grey. Cuttdiff 2 statistical methods were applied to the data and a q value was generated to determine the FDR-adjusted p-value of the test statistic using a Benjamini-Hochberg correction for multiple-testing.

gene_id	gene	locus	sample_1	sample_2	value_1	value_2	log2(fold_change)	test_stat	p_value	q_value	significant
FBgn0001256	ImpL1	3L:13375856-13377099	12CS_CNS	12OR_CNS	0.209048	0.125118	-0.740548	0	1	1	no
FBgn0002573	sens	3L:13389328-13394225	12CS_CNS	12OR_CNS	0.160433	0.188776	0.234709	0	1	1	no
FBgn0003034	Acp70A	3L:13294734-13295022	12CS_CNS	12OR_CNS	0.166839	0.187009	0.164656	0	1	1	no
FBgn0011844	tRNA:D2:69F	3L:13221788-13272742	12CS_CNS	12OR_CNS	0	0	0	0	1	1	no
FBgn0012009	tRNA:V4:70BCa	3L:13422530-13428329	12CS_CNS	12OR_CNS	0	0	0	0	1	1	no
FBgn0012010	tRNA:V4:70BCb	3L:13422530-13428329	12CS_CNS	12OR_CNS	0	0	0	0	1	1	no
FBgn0023095	caps	3L:13221788-13272742	12CS_CNS	12OR_CNS	17.0397	16.4349	-0.0521359	-0.100059	0.8667	0.921151	no
FBgn0036348	CG17687	3L:13342325-13347949	12CS_CNS	12OR_CNS	0.105503	0.0402133	-1.39153	0	1	1	no
FBgn0036350	CG14111	3L:13372391-13374443	12CS_CNS	12OR_CNS	0.0765975	0.0815177	0.0898154	0	1	1	no
FBgn0036351	CG14107	3L:13372391-13374443	12CS_CNS	12OR_CNS	0.0998795	0.0686044	-0.541887	0	1	1	no
FBgn0036352	CG14110	3L:13377890-13379025	12CS_CNS	12OR_CNS	0.066613	0.0665729	-0.000868881	0	1	1	no
FBgn0036353	CG10171	3L:13379045-13380969	12CS_CNS	12OR_CNS	10.3786	10.0216	-0.0504996	-0.0828425	0.88635	0.933465	no
FBgn0036354	CG10191	3L:13381392-13383088	12CS_CNS	12OR_CNS	0.917949	0.981992	0.0972972	0.102339	0.87755	0.927949	no
FBgn0036356	CG10222	3L:13404628-13405553	12CS_CNS	12OR_CNS	6.16855	6.24205	0.0170887	0.0274897	0.9704	0.983085	no
FBgn0036359	CG14105	3L:13419061-13419736	12CS_CNS	12OR_CNS	1.05294	0.293607	-1.84247	-1.51547	0.10185	0.201299	no
FBgn0036360	CG10713	3L:13422530-13428329	12CS_CNS	12OR_CNS	13.6228	10.9772	-0.311518	-0.529466	0.3474	0.485312	no
FBgn0036361	CG10154	3L:13428779-13430233	12CS_CNS	12OR_CNS	0	0	0	0	1	1	no
FBgn0036362	CG10725	3L:13431055-13432109	12CS_CNS	12OR_CNS	0	0	0	0	1	1	no
FBgn0036364	CG14109	3L:13434296-13435520	12CS_CNS	12OR_CNS	0.627366	8.32716	3.73045	4.05092	0.00015	0.000683354	yes
FBgn0036365	CG10732	3L:13435706-13443463	12CS_CNS	12OR_CNS	6.59814	6.10494	-0.112084	-0.17569	0.7684	0.855527	no
FBgn0036366	CG10133	3L:13435706-13443463	12CS_CNS	12OR_CNS	13.3193	12.3536	-0.108586	-0.101144	0.8604	0.917391	no
FBgn0036367	CG10116	3L:13443869-13458639	12CS_CNS	12OR_CNS	0.564427	0.413575	-0.448639	-0.130368	0.84285	0.90591	no
FBgn0036368	CG10738	3L:13443869-13458639	12CS_CNS	12OR_CNS	2.24394	1.51204	-0.569532	-0.871188	0.14765	0.262898	no
FBgn0036369	CG10089	3L:13459926-13467643	12CS_CNS	12OR_CNS	34.6082	31.1742	-0.150759	-0.272244	0.6319	0.752432	no
FBgn0036372	CG10083	3L:13476632-13479398	12CS_CNS	12OR_CNS	22.5715	19.2715	-0.228031	-0.444108	0.46955	0.608788	no
FBgn0036373	CG10741	3L:13479903-13495504	12CS_CNS	12OR_CNS	17.6348	16.7674	-0.0727719	-0.127111	0.8271	0.89557	no
FBgn0036374	CG17689	3L:13495839-13503200	12CS_CNS	12OR_CNS	4.43707	4.86482	0.132779	0.226262	0.69665	0.803255	no
FBgn0036376	CG10743	3L:13506353-13512701	12CS_CNS	12OR_CNS	9.5655	9.22802	-0.0518184	-0.0914778	0.8816	0.930449	no
FBgn0036377	CG10710	3L:13506353-13512701	12CS_CNS	12OR_CNS	1.19585	0.8396	-0.510258	-0.255624	0.66495	0.77883	no
FBgn0036379	bru-3	3L:13531017-13659849	12CS_CNS	12OR_CNS	21.8537	14.3343	-0.608403	-1.04148	0.0711	0.15082	no
FBgn0040813	Nplp2	3L:13349632-13350328	12CS_CNS	12OR_CNS	221.492	100.164	-1.14489	-2.58031	0.00635	0.0195598	yes
FBgn0040814	CG14113	3L:13322317-13322805	12CS_CNS	12OR_CNS	0.0784424	0.208593	1.41098	0	1	1	no
FBgn0052119	CG32119	3L:13221788-13272742	12CS_CNS	12OR_CNS	0.0974037	0.0596398	-0.707703	0	1	1	no
FBgn0052121	CG32121	3L:13410502-13413194	12CS_CNS	12OR_CNS	0.0933487	0.260779	1.48212	0	1	1	no
FBgn0052123	tRNA:CR32123:Psi	3L:13272875-13272928	12CS_CNS	12OR_CNS	0	0	0	0	1	1	no
FBgn0052126	tRNA:CR32126	3L:13205464-13205536	12CS_CNS	12OR_CNS	0	0	0	0	1	1	no
FBgn0052127	tRNA:CR32127	3L:13207208-13207333	12CS_CNS	12OR_CNS	0	0	0	0	1	1	no
FBgn0067703	mir-289	3L:13531017-13659849	12CS_CNS	12OR_CNS	0	0	0	0	1	1	no

Supplementary Table 2: *dokb* expression in the oenocytes. qPCR from oenocyte tissue was used to test expression of genes in three technical replicates. Ct values are reported for the housekeeping gene, *RpL32*, as well as *per*, *Clk* and *dokb*. *per* and *Clk* are known to be expressed at low levels in the oenocytes and were used as positive controls. *dokb* expression was not detected in two of the replicates.

RpL32	***per***	***Clk***	***dokb***
20.2317	32.5311	31.4419	-
20.2198	32.9758	30.7545	35.7885
20.0487	32.3875	31.0203	-

Supplementary Table 3: Allele frequency at *dokb*'s 1049th nucleotide position. The CS and OR strains used in this paper differ at nucleotide position 1049 by either a C (CS) or A (OR), resulting in an amino acid substitution (Ala→Glu). Both alleles at this position appear in numerous wild-type *Drosophila melanogaster* strains collected across 5 continents. Two strains from Raleigh, NC have a G at this nucleotide position, resulting in a glycine.

Country	Locality	Continent	Elevation	Total Number of Samples	Number of Samples with C	Number of Samples with A	Number of Samples with G
Netherlands	Houten	Europe / N. Africa	4	17	16	1	0
Australia	Sorell TAS	Oceania	18	12	11	1	0
Egypt	Cairo	Europe / N. Africa	25	13	11	2	0
United States	Winters CA	America	41	24	20	4	0
China	Beijing	Asia	52	8	8	0	0
United States	Raleigh NC	America	91	190	168	20	2
France	Lyon	Europe / N. Africa	175	56	40	16	0
Nigeria	Maiduguri	Africa	295	6	5	1	0
Gabon	Franceville	Africa	332	10	9	1	0
United States	Ithaca NY	America	344	16	16	0	0
South Africa	Phalaborwa	Africa	350	23	22	1	0
Ethiopia	Gambella	Africa	525	10	10	0	0
Zambia	Siavonga	Africa	530	198	197	1	0
Guinea	Dondé	Africa	801	7	7	0	0
Zimbabwe	Sengwa	Africa	865	5	5	0	0
Kenya	Marigat	Africa	1062	6	6	0	0
Uganda	Namulonge	Africa	1134	6	6	0	0
Ethiopia	Ziway	Africa	1642	5	5	0	0
Ethiopia	Bonga	Africa	1725	5	5	0	0
South Africa	Barkly East	Africa	1800	5	5	0	0
South Africa	Fouriesburg	Africa	1800	5	5	0	0
Uganda	Kisoro	Africa	1925	5	5	0	0
Rwanda	Gikongoro	Africa	1927	28	28	0	0
South Africa	Dullstroom	Africa	2000	33	33	0	0
Cameroon	Oku	Africa	2169	13	13	0	0
Kenya	Nyahururu	Africa	2303	6	6	0	0
Ethiopia	Dodola	Africa	2492	6	6	0	0
Ethiopia	Debre Birhan	Africa	2840	5	4	1	0
Ethiopia	Fiche	Africa	3070	26	26	0	0
Malawi	Mwanza	Africa	-	5	5	0	0

Supplementary Table 4: The interaction criteria (distance, angle and duration) used to create social networks for each line. The figures that correspond to each social network are listed on the right.

Lines	Distance (body lengths)	Angle (°)	Duration (s)	Figures
CS {*CG14109*$^{+1}$} aka CS{*dokb*$^{+1}$}	1.50	130.00	0.55	Figure 2d, Figure 3, Figure 4d
OR {*CG14109*$^{+2}$} aka OR{*dokb*$^{+2}$}	1.50	105.00	0.35	
OR {*CG14109*$^{+1}$} aka OR{*dokb*$^{+1}$}	1.75	120.00	0.50	
CS {*CG14109*$^{+2}$} aka CS{*dokb*$^{+2}$}	2.00	80.00	0.65	
dokb^{+2}	175.00	125.00	0.45	Figure 4a
dokb^{+2}/Df(3L)ED4502	2.00	120.00	0.93	
dokb^{+2}/*dokb*^{n2}-Gal4	2.00	27.50	0.10	
Df(3L)ED4502/*dokb*^{n2}-Gal4	1.50	15.00	0.25	
WT Control	1.50	125.00	0.60	Figure 4b
UAS Control	1.50	110.00	0.55	
Gal4 Control	2.00	150.00	1.30	
dokb RNAi Knockdown	1.75	145.00	1.35	
dokb^{+1}	2.25	80.00	0.60	Figure4c
dokb^{n1}	2.00	70.00	0.50	
Gal4 Control	1.75	135.00	1.05	Figure 4e
UAS Control	1.50	135.00	1.20	
Nplp2 RNAi Knockdown	1.75	130.00	1.70	
All Introgression Lines	2.00	90.00	1.50	Figure 1a, Suppl. Fig. 2, Suppl. Fig. 3
All Recombinant Lines	2.00	90.00	1.50	

Supplementary Figures

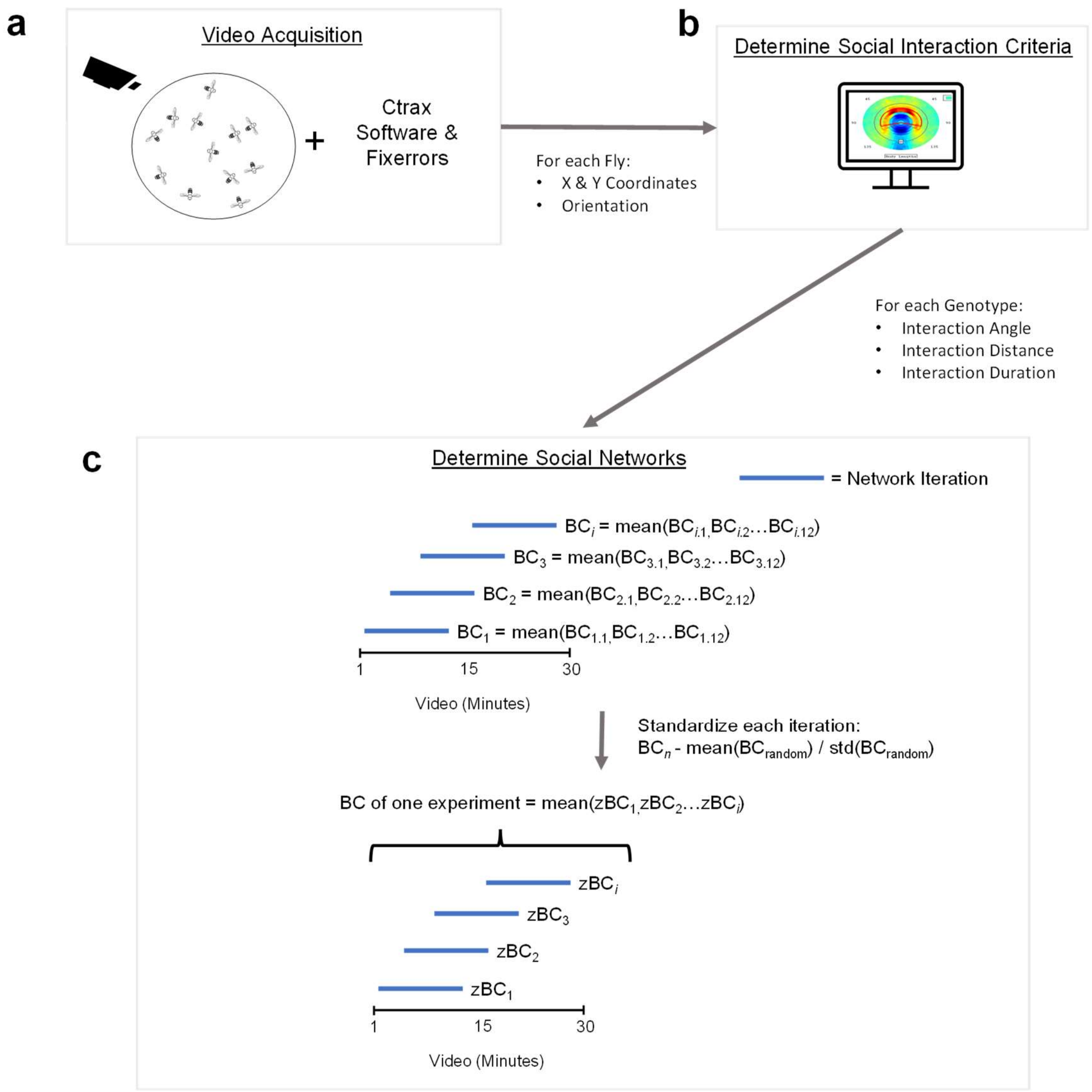

Supplementary Fig. 1: Acquiring and analyzing *Drosophila* social networks. **a**, Twelve male flies were gently aspirated into a circular plexiglass arena and placed under a camera. Flies acclimated undisturbed for 10 min. followed by 30 min. of video recording. Flies were then discarded. The video was processed through Ctrax and potential tracking errors were manually fixed using Fixerrors[52]. The output was a file that contains the identity, position and orientation of each fly for each frame for the entire video. The entirety of step a was repeated independently approximately 20 times for each genotype. **b**, After all videos for a genotype were acquired and processed (step a), the criteria at which a given genotype socially interacts was determined using open-source software[53]. Briefly, this software objectively determines the angle, distance and duration that occurs at a higher frequency than random for each genotype. When these criteria are met by flies in an arena, they are said to be socially interacting. **c**, Social networks were evaluated by calculating iterative networks using a moving-window boxcar filter at 25% network density[16,54]. The first network iteration represents the first 33 unique interactions, the second network iteration ignores the first unique interaction and adds a subsequent unique interaction…the *i*th network iteration ignores the first *i*-1 interactions. For *n* network iterations, the betweenness centrality (BC*n*) was determined by averaging the BC of all twelve flies. Each network iteration was standardized for degree distribution. Briefly, for each iteration, 10 000 random networks with the same in- and out-degree distribution were generated and used to calculate the z score (zBC*n*). The final BC for a given experiment (represented as a single dot on a dot plot graph) was calculated by averaging all network iteration zBC values for a given 30 min. experiment.

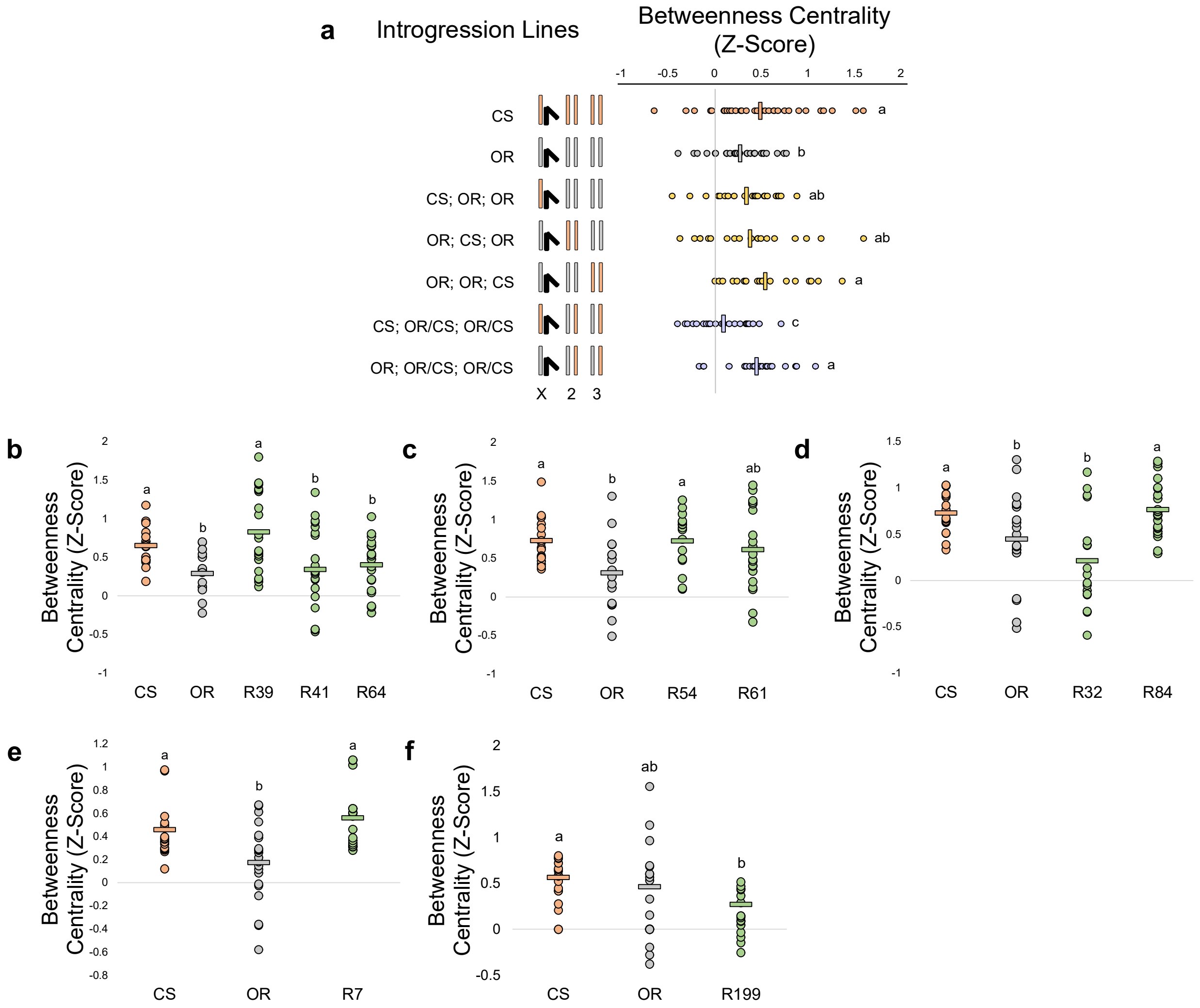

Supplementary Fig. 2: Behavioural phenotypes of recombinant and introgression lines used to map a locus. **a,** Left: A visual representation of introgression lines and the chromosomes for each line tested. CS chromosomes are illustrated in orange, OR chromosomes are illustrated in grey. The chromosomes are labelled on the bottom. Only males are depicted and were used in all behavioural experiments. Right: Betweenness centrality of wild type, introgression lines, and heterozygotes from networks formed by groups of male *Drosophila*. Each dot represents the average betweenness centrality from a network formed by a distinct group of 12 flies. The mean for each genotype is indicated by a vertical line. Wild-type flies (orange and grey), introgression lines (yellow) and heterozygotes (purple) were used in this experiment. A permuted ANOVA was performed, and statistical significance (α=0.05) are indicated by letters as determined by 95% confidence intervals. CS: *n*=32, OR: *n*=27, CS;OR;OR: *n*=22, OR;CS;OR: *n*=19, OR;OR;CS: *n*=19, CS;CS/OR;CS/OR *n*=22, OR;CS/OR;CS/OR: *n*=22. Permuted p-value<0.05. **b-f,** Betweenness centrality was calculated from the social networks of different groups of flies. Each dot represents the average betweenness centrality from a network formed by a distinct group of 12 flies. Averages for each genotype are shown by a horizontal line. A permuted ANOVA was performed, and statistical significance (α=0.05) are indicated by letters, as determined by 95% confidence intervals. **b,** Line R39's (*n*=22) mean betweenness centrality is statistically indistinguishable from CS (*n*=15), while lines R41's (*n*=21) and R64's (*n*=20) betweenness centrality is statistically indistinguishable from OR (*n*=17). Permuted p-value<0.001. **c,** Line R54's (*n*=17) betweenness centrality is statistically indistinguishable from CS (*n*=18), while line R61's (*n*=20) betweenness centrality is statistically similar to both CS and OR (*n*=16). Permuted p-value<0.05. **d**, Line R32's (*n*=17) betweenness centrality is statistically indistinguishable from OR (*n*=19), while line R84's (*n*=22) betweenness centrality is statistically indistinguishable from CS (*n*=17). Permuted p-value<0.001. **e**, Line R7's (*n*=16) betweenness centrality is statistically indistinguishable from CS (*n*=21) and different from OR (*n*=19).Permuted p-value<0.001. **f,** Line R199's (*n*=21) betweenness centrality is statistically similar to OR (*n*=15) and different from CS (*n*=16). Permuted p-value<0.05.

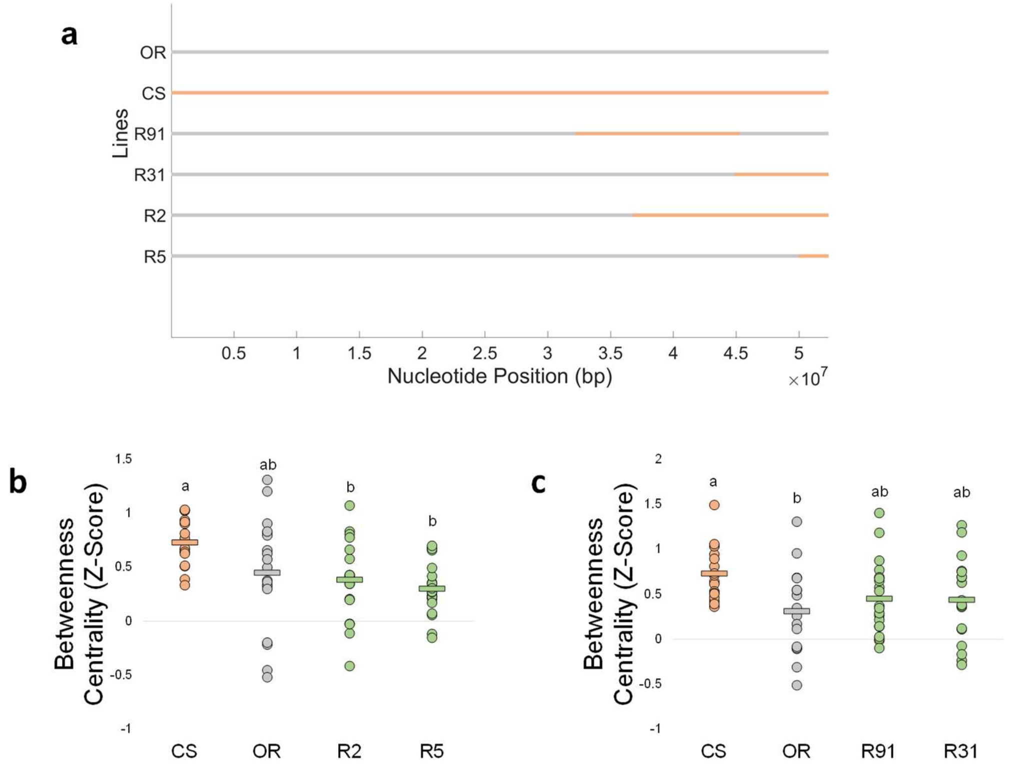

Supplementary Fig. 3: Recombination events outside the 474 kb locus show expected betweenness centrality phenotypes. a, Visual representation of recombination events on the 3rd chromosome in various lines of *Drosophila* (x-axis) as determined by SNP genotyping. OR regions are displayed in grey, CS regions are displayed in orange. Nucleotide position (bp) of the 3rd chromosome is on the x-axis. **b-c** . Each dot represents the average betweenness centrality from a network formed by a distinct group of 12 flies. Averages for each genotype are shown by a horizontal line. Statistical significance is indicated by letters and was determined by a one-way ANOVA, followed by a Tukey-Kramer post hoc test. **b,** Lines R2 and R5 form networks with the same betweenness centrality as OR flies. CS: *n*=17, OR: *n*=19, R2: *n*=18, R5: *n*=20. $F_{(3,75)}=5.02$, $p<0.05$. **c,** Lines R91 and R31 form networks with the same betweenness centrality as OR flies. CS: *n*=18, OR: *n*=16, R91: *n*=20, R31: *n*=18. $F_{(3,71)}=3.13$, $p<0.05$.

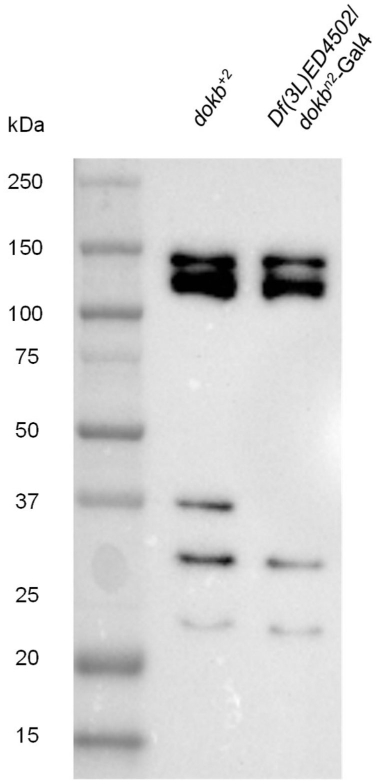

Supplementary Fig. 4: Western blot of *dokb* null line and wild-type control. Western blot of proteins extracted from the heads of OR (*dokb*$^{+2}$) and *dokb* null flies (*Df*(3L)ED4502/*dokb*^{n2}-Gal4). 1° Ab=Rabbit anti-CG14109_AA_61-74 (1:1000). 2° Ab=Donkey ECL Anti-Rabbit IgG (1:20,000). A missing protein band in *dokb* null flies is found at approximately 37 kDa.

```
CLUSTAL O(1.2.4) multiple sequence alignment


CS_DOKB      MVNELNRPQNGDNAALSERLAASNRQLQEMQEEHRQLLEEMETLRLRAAELTLLNAQRRQ  60
OR_DOKB      MVNELNRPQNGDNAALSERLAASNRQLQEMQEEHRQLLEEMETLRLRAAELTLLNAQRRQ  60
             ************************************************************
CS_DOKB      VRQSTEEEEVQSHVESSSETVTVASSASASGESTSREEAPSGEEEGEEDKEEQASYLQAK  120
OR_DOKB      VRQSTEEEEVQSHVESSSETVTVASSASASGESTSREEAPSGEEEGEEDKEEQASYLQAK  120
             ************************************************************
CS_DOKB      LNEIANLKAQFKRVQNMVDTTKMIEEHMSSRQTVQVQSSTSVQTSRQTTSSEVRVASEAV  180
OR_DOKB      LNEIANLKEQFKRVQNMVDTTKMIEEHMSSRQTVQVQSSTSVQTSRQTTSSEVRVASEAV  180
             ******** ***************************************************
CS_DOKB      ESAQEENPSTSSSAPDNAELLNSMLNMFTDFTSDLRGQAEGLRAERDRIRALKEDIIQRK  240
OR_DOKB      ESAQEENPSTSSSAPDNAELLNSMLNMFTDFTSDLRGQAEGLRAERDRIRALKEDIIQRK  240
             ************************************************************
CS_DOKB      QGK      243
OR_DOKB      QGK      243
```

Supplementary Fig. 5: Alignment of predicted DOKB protein sequences of CS and OR strains used in our experiments. Highlighted amino acids are different between the two wild-type strains.

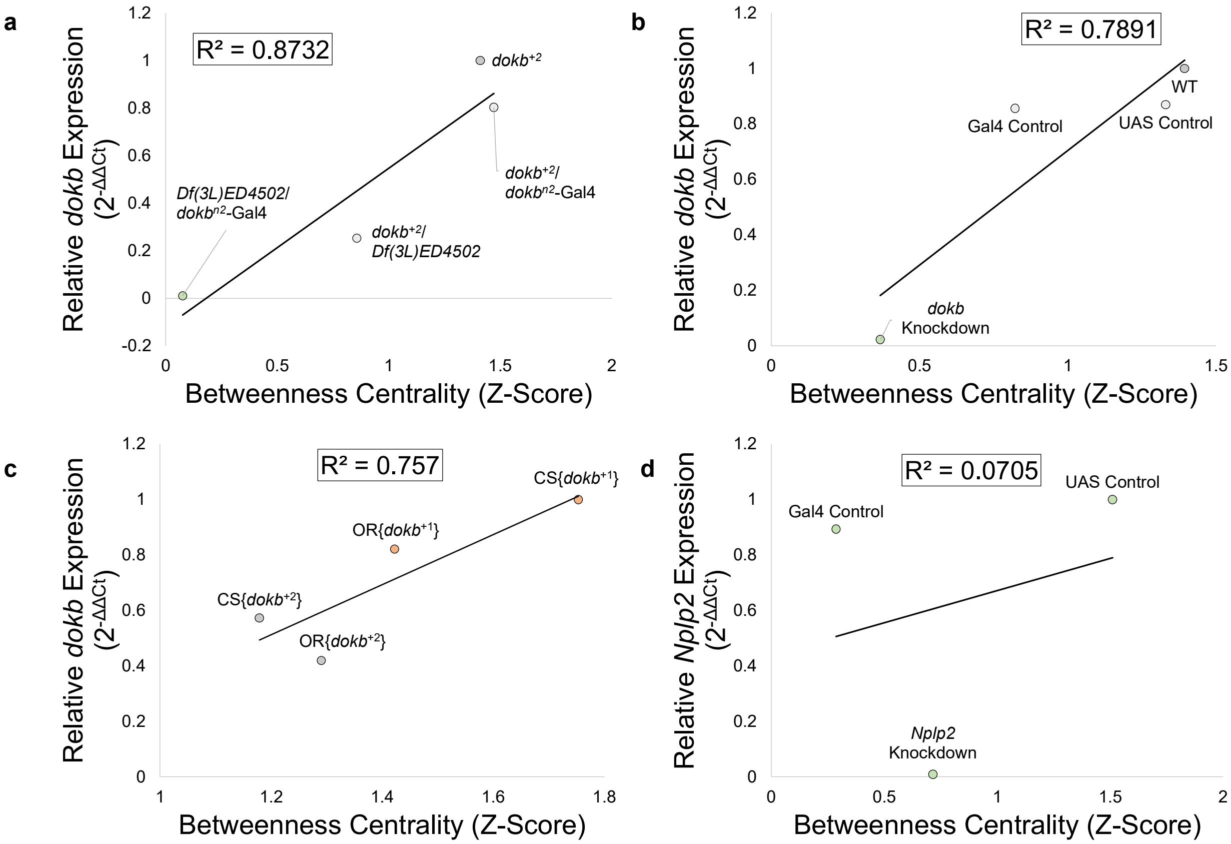

Supplementary Fig. 6: *dokb* RNA expression correlates to betweenness centrality. A line of best fit was plotted, and the coefficient of determination (R^2) was calculated. Data used here are presented in Figure 4 (see Figure 4 legend for sample sizes) **a,** *dokb* expression correlates with the betweenness centrality of networks formed by genotypes containing two copies of *dokb* (*dokb*$^{+2}$), one copy of *dokb* (*dokb*$^{+2}$/*Df*(3L)ED4502 and *dokb*$^{+2}$/*dokb*^{n2}-Gal4) and no copies of *dokb* (*Df*(3L)ED4502/ *dokb*^{n2}-Gal4). R^2= 0.8732. **b,** *dokb* expression correlates with betweenness centrality of networks formed by control lines and a *dokb* RNAi knockdown line. R^2=0.7891. **c,** The level of *dokb* expression correlates to the *dokb* allele, and not the genetic background. R^2=0.757. **d,** *Nplp2* expression does not correlate to betweenness centrality in *Nplp2* knockdown flies and its controls. R^2=0.0705.

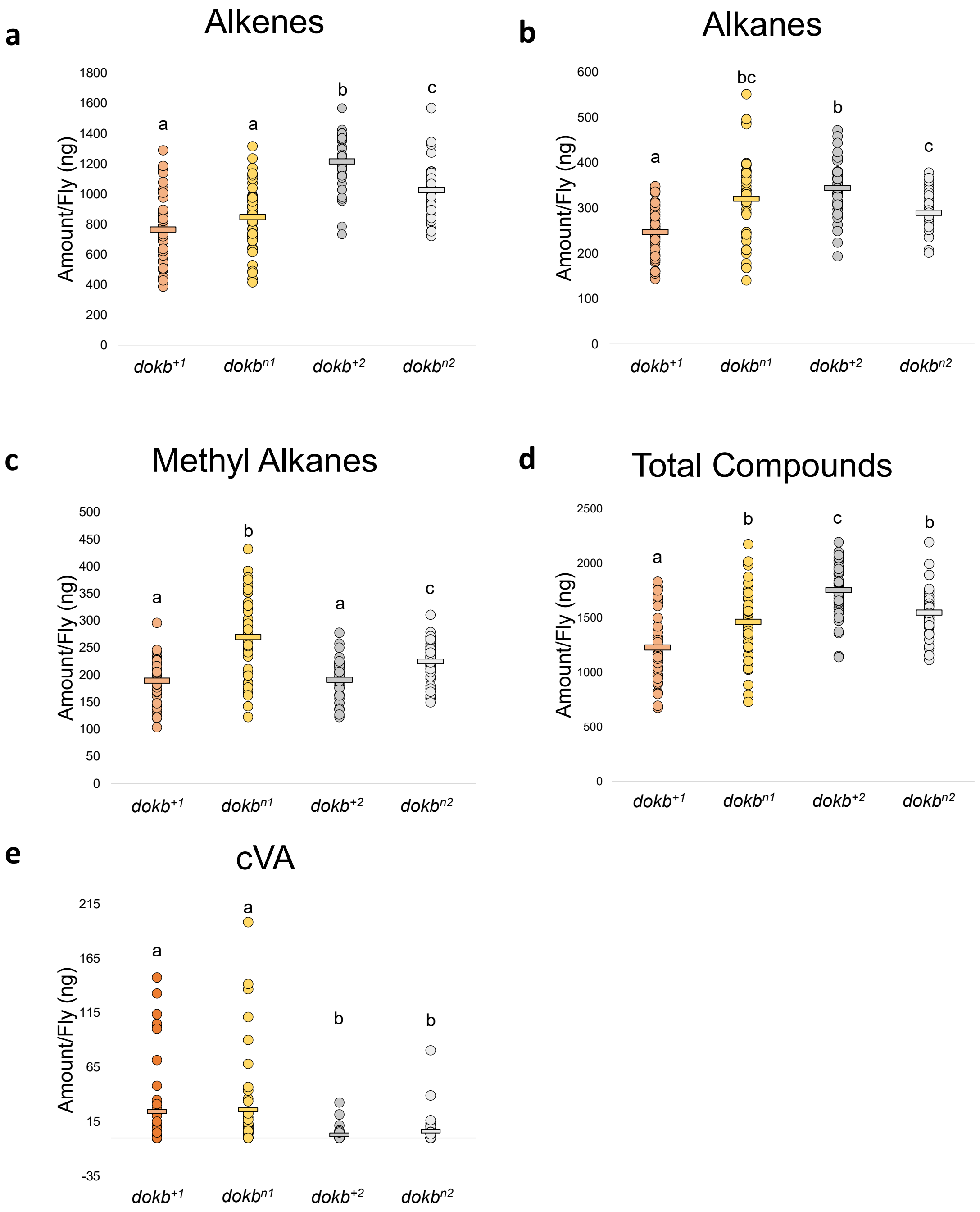

Supplementary Fig. 7: Hydrocarbons of wild type and *dokb* null lines. Dots represent amounts of hydrocarbon on an individual fly. Horizontal bars represent mean hydrocarbon amounts for each genotype. Statistical significance is indicated by letters and was determined by a one-way ANOVA followed by a Tukey-Kramer post hoc test. Wild-type CS ($dokb^{+1}$) and OR ($dokb^{+2}$) flies were compared to CS flies with a deleted *dokb* coding sequence ($dokb^{n1}$) and to OR flies with a deleted *dokb* coding sequence ($dokb^{n2}$). **a,** Average amount of alkenes $F_{(3,171)}=46.26$, $p<0.001$. **b,** Average amount of alkanes. $F_{(3,171)}=18.28$, $p<0.001$. **c,** Average amount of methyl alkanes $F_{(3,171)}=25.28$, $p<0.001$. **d,** Average amount of total hydrocarbons. $F_{(3,171)}=27.99$, $p<0.001$. **e,** Average amount of cVA. $F_{(3,171)}=6.72$, $p<0.001$. $dokb^{+1}$: n=45, $dokb^{n1}$: n=45, $dokb^{+2}$: n=44, $dokb^{n2}$: n=41.

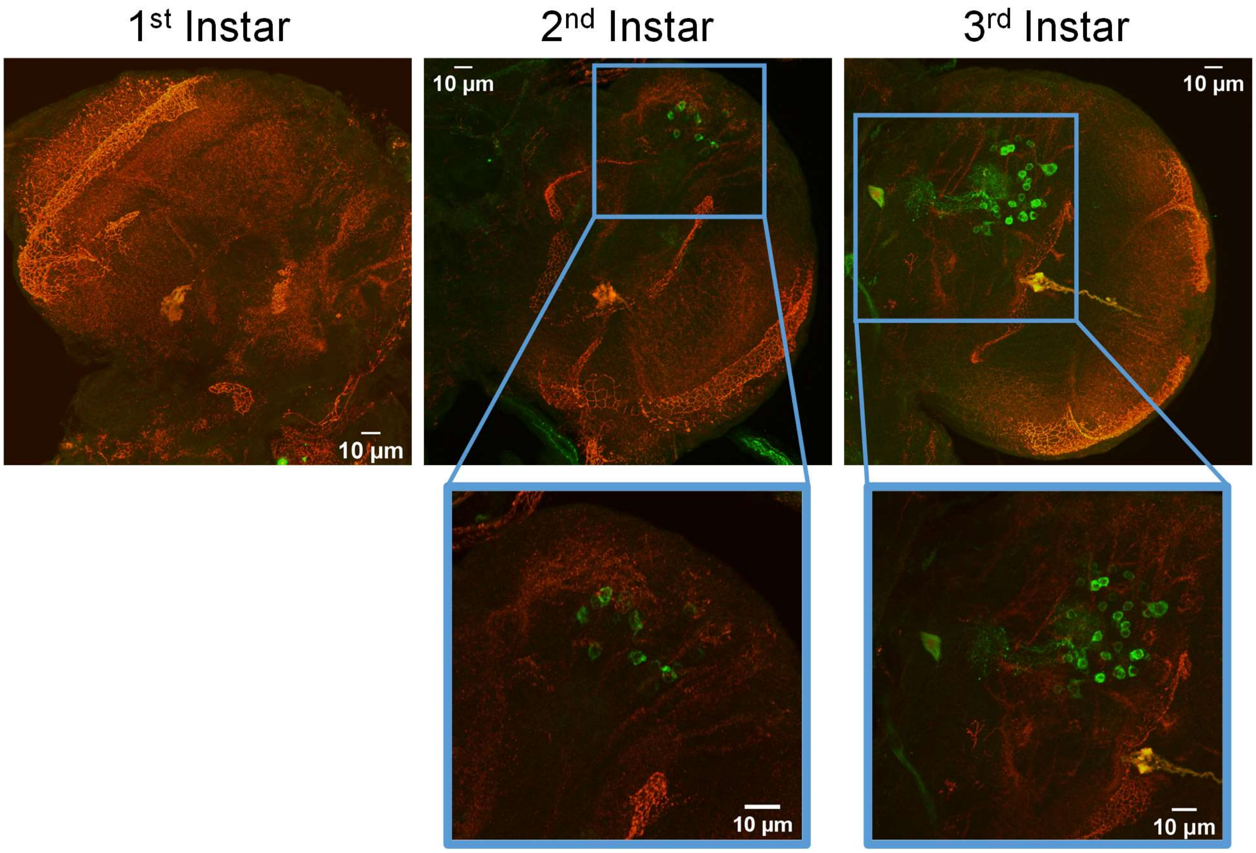

Supplementary Fig. 8: Expression of *dokb* in a larval central brain lobe using *dokb*^{n2}-Gal4 line and a GFP reporter. *dokb*^{n2}-Gal4 expression patterns reported by UAS-mCD8::GFP reporter (green) in a single central brain lobe of the 1st, 2nd and 3rd instar larval brain (left to right). Zoomed in images are shown with a blue outline. Scale bars are as indicated in the corners. Counterstained with anti-DN-cad antibody (red). n≥3

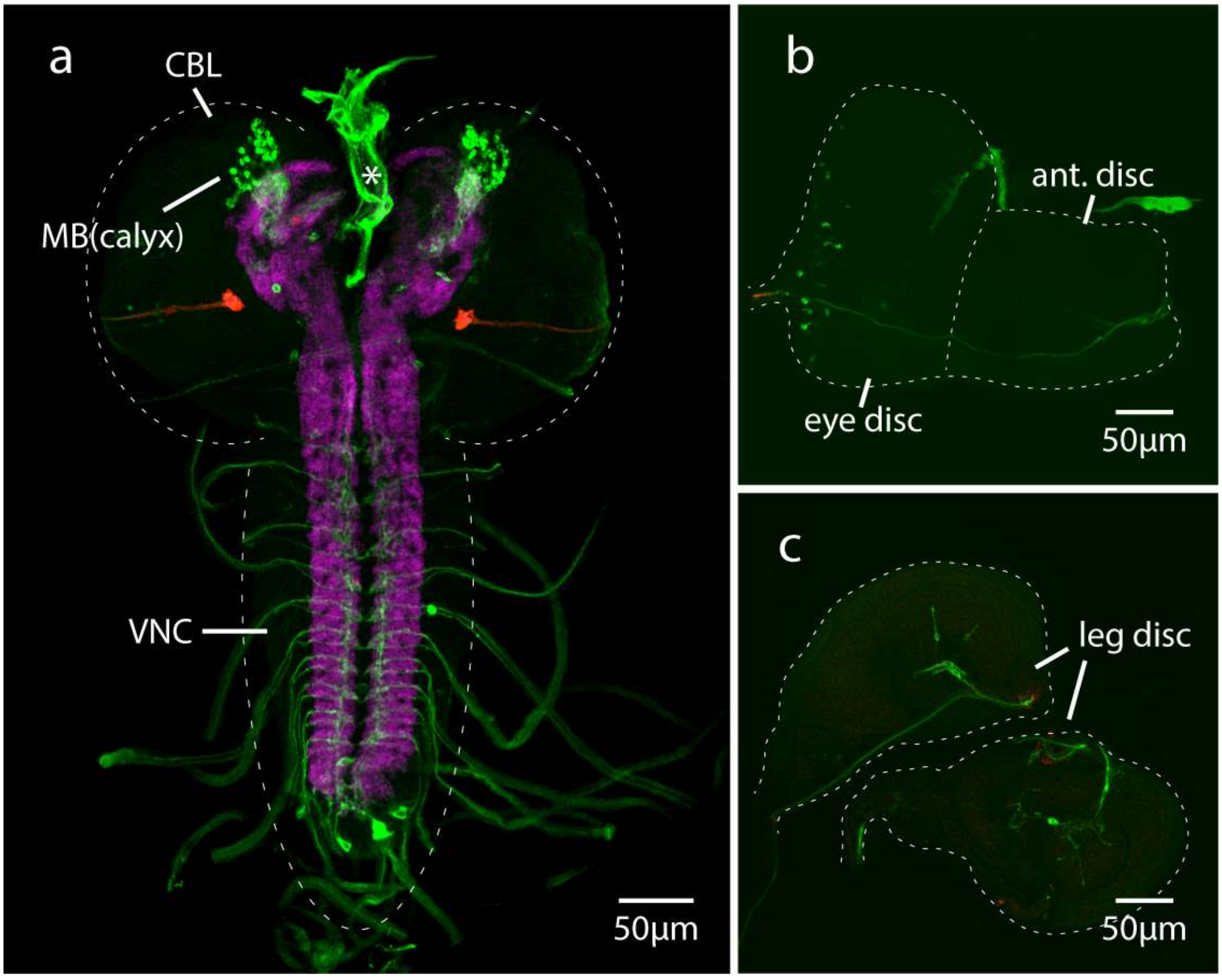

Supplementary Fig. 9: Expression of *dokb* using *dokb^{n2}*-Gal4 line and a GFP reporter in the larval CNS. *dokb^{n2}*-Gal4 expression patterns reported by UAS-mCD8.GFP (green). Immunostained with anti-nc82 antibody (magenta). Scale bars are indicated at the bottom. **a,** *dokb* expression in the larval brain and ventral nerve cord; CBL=central brain lobe, MB (calyx)=mushroom body calyx, VNC=ventral never cord, *= tissue with an asterisk is artifact labelling of the esophagus. **b,** *dokb* expression in the eye disc and antennal disc. **c,** *dokb* expression in the leg discs. n≥3

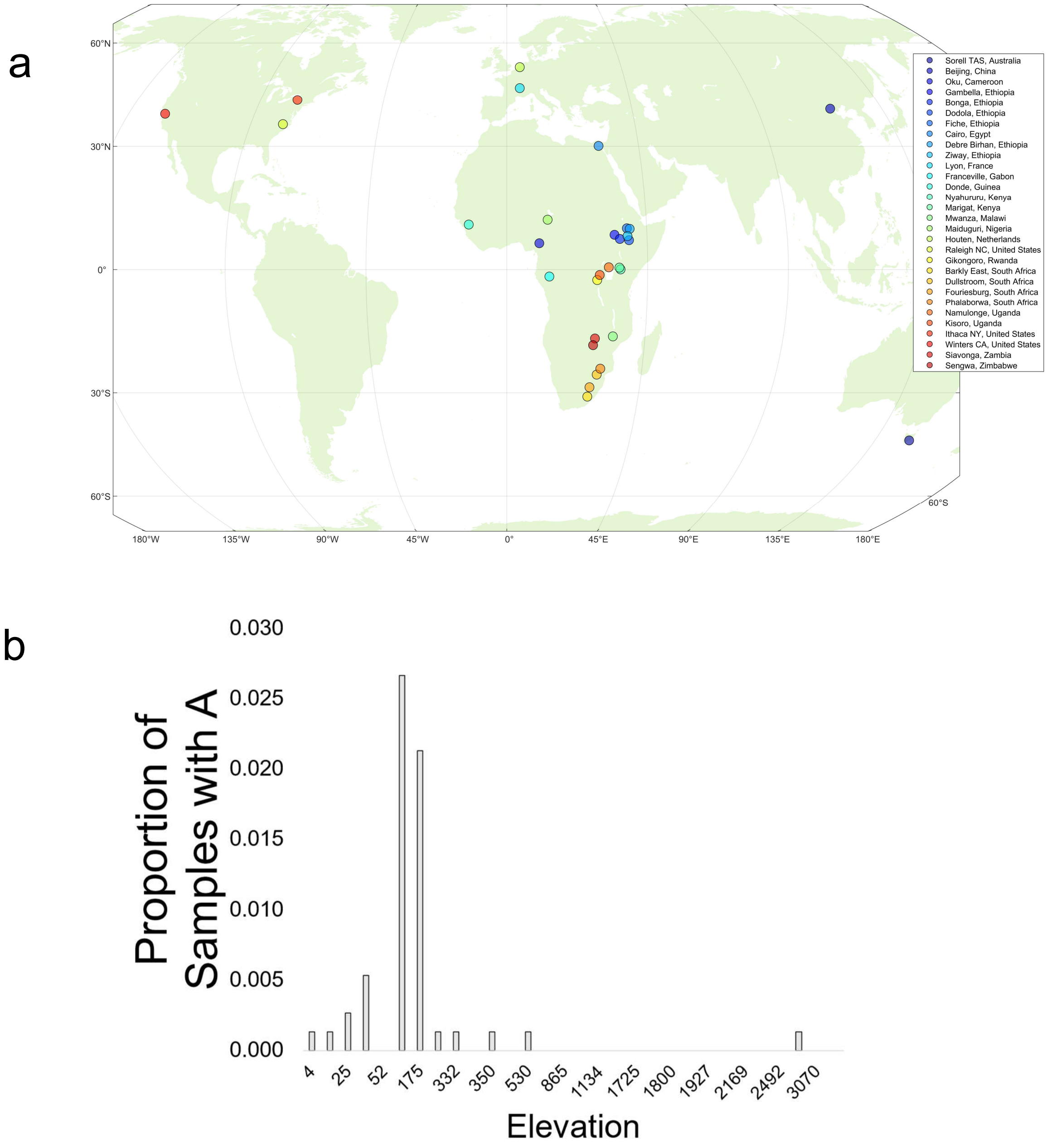

Supplementary Fig. 10: Investigating the *dokb* alleles in natural populations of *Drosophila melanogaster*. Sequence and location data were acquired from PopFly (https://popfly.uab.cat/). **a,** A world map showing the locations from which the *Drosophila melanogaster* samples were collected. Map was created in Matlab using geoplot (https://www.mathworks.com/help/matlab/ref/geoplot.html). **b,** The proportion of samples at each elevation that have adenosine (A) at nucleotide position 1049 of their *dokb* sequence.
