# Supplementary material for: The gene “degrees of kevin bacon” (dokb) regulates a social network behaviour in Drosophila melanogaster
Source: Nat Commun. 2024 Apr 30;15:3339. doi: 10.1038/s41467-024-47499-8 (PMC11061139; doi:10.1038/s41467-024-47499-8)
Supplement: Supplementary file 3 — Reporting Summary [file 41467_2024_47499_MOESM3_ESM.pdf]

nature portfolio

Corresponding author(s): Joel Levine

Last updated by author(s): Mar 5, 2024

Reporting Summary

Nature Portfolio wishes to improve the reproducibility of the work that we publish. This form provides structure for consistency and transparency in reporting. For further information on Nature Portfolio policies, see our Editorial Policies and the Editorial Policy Checklist.

Statistics

For all statistical analyses, confirm that the following items are present in the figure legend, table legend, main text, or Methods section.

n/a	Confirmed	
☐	☒	The exact sample size (*n*) for each experimental group/condition, given as a discrete number and unit of measurement
☐	☒	A statement on whether measurements were taken from distinct samples or whether the same sample was measured repeatedly
☐	☒	The statistical test(s) used AND whether they are one- or two-sided<br>*Only common tests should be described solely by name; describe more complex techniques in the Methods section.*
☐	☒	A description of all covariates tested
☐	☒	A description of any assumptions or corrections, such as tests of normality and adjustment for multiple comparisons
☐	☒	A full description of the statistical parameters including central tendency (e.g. means) or other basic estimates (e.g. regression coefficient) AND variation (e.g. standard deviation) or associated estimates of uncertainty (e.g. confidence intervals)
☐	☒	For null hypothesis testing, the test statistic (e.g. *F*, *t*, *r*) with confidence intervals, effect sizes, degrees of freedom and *P* value noted<br>*Give P values as exact values whenever suitable.*
☒	☐	For Bayesian analysis, information on the choice of priors and Markov chain Monte Carlo settings
☒	☐	For hierarchical and complex designs, identification of the appropriate level for tests and full reporting of outcomes
☒	☐	Estimates of effect sizes (e.g. Cohen's *d*, Pearson's *r*), indicating how they were calculated

Our web collection on statistics for biologists contains articles on many of the points above.

Software and code

Policy information about availability of computer code

Data collection	Fly trajectories were determined using CTRAX (v. 0.5.13).
Data analysis	Social network and statistical analyses were performed using MATLAB (MathWorks, v2014a). The genetic mapping social network experiments were performed using code described in Schneider, Dickinson & Levine (2012) with no deviations. All other network experiments in this study were performed as described in Schneider, Dickinson & Levine (2012) with the following deviations: networks were generated using the method described in Schneider & Levine (2014) to determine the social interaction criteria. All code from the Levine Lab are available upon request.

For manuscripts utilizing custom algorithms or software that are central to the research but not yet described in published literature, software must be made available to editors and reviewers. We strongly encourage code deposition in a community repository (e.g. GitHub). See the Nature Portfolio guidelines for submitting code & software for further information.

Data

Policy information about availability of data

All manuscripts must include a data availability statement. This statement should provide the following information, where applicable:

- Accession codes, unique identifiers, or web links for publicly available datasets
- A description of any restrictions on data availability
- For clinical datasets or third party data, please ensure that the statement adheres to our policy

Source data is available as a source data file. This file contains all behavioural, hydrocarbon and qPCR data generated in this study. The raw RNAseq data have been deposited in the NCBI's SRA database under accession code PRJNA1082663 (https://www.ncbi.nlm.nih.gov/sra/PRJNA1082663). Natural variant data from strains of Drosophila melanogaster were acquired from the PopFly database (https://popfly.uab.cat/).

Research involving human participants, their data, or biological material

Policy information about studies with human participants or human data. See also policy information about sex, gender (identity/presentation), and sexual orientation and race, ethnicity and racism.

Reporting on sex and gender	N/A
Reporting on race, ethnicity, or other socially relevant groupings	N/A
Population characteristics	N/A
Recruitment	N/A
Ethics oversight	N/A

Note that full information on the approval of the study protocol must also be provided in the manuscript.

Field-specific reporting

Please select the one below that is the best fit for your research. If you are not sure, read the appropriate sections before making your selection.

☐ Life sciences ☐ Behavioural & social sciences ☒ Ecological, evolutionary & environmental sciences

For a reference copy of the document with all sections, see nature.com/documents/nr-reporting-summary-flat.pdf

Ecological, evolutionary & environmental sciences study design

All studies must disclose on these points even when the disclosure is negative.

Study description	We mapped a gene required for the expression of the social network property, betweenness centrality, in Drosophila. We verified the gene using CRISPR/Cas9, characterized its effects on cuticular hydrocarbon expression and its neuroanatomical expression pattern using a Gal4 insertion at the gene's locus. For the network data, n was approximately 20/treatment or genotype. Outliers were removed as described in the Methods section. For qPCR, 3 technical replicates were run for each sample and n= at least 2/genotype. For RNAseq, n=3 biological replicates. For hydrocarbon analysis, we extracted and analyzed the hydrocarbons from 40-50 individual flies/genotype. All experiments were analyzed using a one way ANOVA, except when comparing only two groups, in which case a t-test was performed. A Tukey-Kramer post hoc test was performed after significance was found in an ANOVA.
Research sample	The network behavioural experiments were performed on groups of 12 male Drosophila melanogaster flies of various strains. The Canton-S (CS) and Oregon-R (OR) strains represent two different wildtype variants of Drosophila melanogaster. Behavioural experiments were performed on 3-day old adult male flies housed with food in a 12 hr light-dark incubator with 60% humidity at 25C.
Sampling strategy	Sample sizes for behavioural data was approximately 20. This sample size was chosen based on previous studies [(Schneider, Dickinson & Levine (2012)), (Rooke et al. (2020)), (Alwash et al. (2021)), (Jezovit et al. (2020)]. Cuticular hydrocarbon sample size was 40-50 individuals/genotype. These sample sizes were chosen based on previous studies [(Krupp et al. (2008)), (Kent et al. (2007)), (Billeter et al. (2009)), (Krupp et al. (2013))]. qPCR samples had 3 technical replicates and at least 2 biological replicates. These sample sizes were chosen based on previous studies [(Krupp et al. (2008)) and (Krupp et al. (2013)).
Data collection	Behavioural data was collected as was previously described in Schneider, Dickinson & Levine (2012) and were collected by R. Rooke, M. Golemiec and A. Rasool. Briefly, 30 min. fly videos were filmed using a FireflyMV camera (Point Gray) and trajectories were obtained by running the videos through Ctrax (v 0.5.13) and manually inspected/fixed for errors. Immunohistochemisty images were acquired by J. Krupp. All CNS images were obtained from a Zeiss LSM880 microscope and muscular tissue images were obtained from a Zeiss LSM880 microscope.

Timing and spatial scale	Recombinant mapping began in 2012 and 2016. The rest of the data were collected between 2016-2023.
Data exclusions	As has been previously done [see Rooke et al. (2020), Alwash et al. (2021), Jezovit et al. (2020)], for all network experiments, outliers ≥75th quartile+(1.5 × IQR) or ≤25th quartile-(1.5 × IQR) were removed before statistical testing.
Reproducibility	Reproducability was determined by using CS and OR wildtype controls. Every time we graph a network experiment, we plot ~20 independent replicates. For every plot of these replicates, we run behavioural controls by comparing CS to OR. In these experiments, when the expected difference between CS and OR is not statistically significant, we exclude the entire data set. All attempts at replication were not successful and the experiment reported in Fig. 2e had to be repeated due to failure of these behavioral controls.
Randomization	Flies were randomly chosen from a vial to run in our behavioural experiments. All experiments were randomized.
Blinding	Blinding was not performed because we relied on methods from machine vision to analyze our behavioural data.

Did the study involve field work? ☐ Yes ☒ No

Reporting for specific materials, systems and methods

We require information from authors about some types of materials, experimental systems and methods used in many studies. Here, indicate whether each material, system or method listed is relevant to your study. If you are not sure if a list item applies to your research, read the appropriate section before selecting a response.

Materials & experimental systems

n/a	Involved in the study	
☐	☒	Antibodies
☒	☐	Eukaryotic cell lines
☒	☐	Palaeontology and archaeology
☐	☒	Animals and other organisms
☒	☐	Clinical data
☒	☐	Dual use research of concern
☒	☐	Plants

Methods

n/a	Involved in the study	
☒	☐	ChIP-seq
☒	☐	Flow cytometry
☒	☐	MRI-based neuroimaging

Antibodies

Antibodies used	Unique antibodies were generated specifically for this study. Antibodies were raised in New Zealand rabbits against the synthesized peptide VRQSTEEEEVQSHV, which corresponds to amino acid positions 61-74 in the CG14109 protein. The resulting antibody preparation was termed anti-CG14109_AA_61-74. All peptide synthesis and antibody production were performed by GenScript (Piscataway, NJ, USA) using the PolyExpress polyclonal antibody express service.<br><br>For detecting dokb-GAL4 expression across larval development in a central brain lobe, larval CNS samples were fixed with 4% paraformaldehyde for 20 min. at RT. The 1° antibody, rabbit anti-GFP (Cell Signaling Technology), was applied at 1:1000 and the counterstaining antibody, mouse anti-DN-cad (Cell Signaling Technology), was applied at a 1:5 concentration. Alex Fluor 488 Donkey Anti-Rb IgG (H+L) (Invitrogen) and Alex Fluor 555 Donkey Anti-Rb IgG (H+L) (Invitrogen) were applied at a 1:500 concentration.<br><br>For detecting dokb-GAL4 expression in the larval and adult CNS, samples were fixed in 4% paraformaldehyde for 20 min. at RT. Samples were labelled with rabbit anti-GFP.Alexa 488 conjugate (1:400; Invitrogen) and counterstained with primary mouse anti-Brp (nc82) (1:40; DSHB) and secondary donkey anti-mouse Alexa 647 (1:400; Invitrogen).
Validation	See Supplementary Figure 4 of this manuscript for validation of the dokb-specific antibody. The rabbit-anti GFP primary antibody was validated by Cell Signaling Technology (https://www.cellsignal.com/products/primary-antibodies/gfp-d5-1-rabbit-mab/2956). Mouse anti-DN-cad antibody was verified by Cell Signaling Technology (https://www.cellsignal.com/products/primary-antibodies/n-cadherin-13a9-mouse-mab/14215). Rabbit anti-GFP.Alexa 488 conjugate antibody was validated by Invitrogen (https://www.thermofisher.com/antibody/product/GFP-Antibody-Polyclonal/A-21311). Validation of the mouse anti-Brp antibody can be found at https://dshb.biology.uiowa.edu/product/1401.

Animals and other research organisms

Policy information about studies involving animals; ARRIVE guidelines recommended for reporting animal research, and Sex and Gender in Research

Laboratory animals	Canton-S (CS) and Oregon-R (OR) wildtype flies were obtained from J. C. Hall (Emeritus at Brandeis University, Waltham, MA). Introgression and recombinant lines were generated through a series of crosses from these wildtype lines. Recombinant lines had OR as their X and 2nd chromosome and an OR recombined with CS as their 3rd chromosome.<br><br>+;+;dokbn2-Gal4, dokbn1, CS(dokb+2), dokbn2 and OR(dokb+1) were generated using CRISPR/Cas9 in collaboration with

WellGenetics Inc. Lines were verified with DNA sequencing.
dokb null lines (dokbn1, dokbn2 and dokbn2-Gal4) were generated such that the 980bp coding sequence (+16 nt from ATG to -85 nt from stop codon of dokb) was deleted and replaced with a Stop-RFP cassette with 3-frames of stop codons and a 3XP3-RFP, except for the dokbn2-Gal4 line which was replaced by a T2A-Gal4::VP16 cassette and 3XP3-RFP.

For the CS(dokb+2) and OR(dokb+1) swap lines, the entire gene region of dokb, from the promoter to the 3'UTR (1960bp) in one strain was deleted and replaced by the gene region from the other strain with an inverted PBacDsRed marker inserted into the 2nd intron of dokb. The marker was excised before lines were used in behavioural experiments.

The CG14109 deficiency null was generated by selecting progeny from crossing virgin +;+;dokbn2-Gal4 females to deficiency males (w[1118]; Df(3L)ED4502, P{w[+mW.Scer\FRT.hs3]=3'.RS5+3.3'}ED4502/TM6C, cu[1] Sb[1]; Bloomington Stock Center Line #8097). The Nplp2 RNAi knockdown flies were generated by selecting progeny from crossing Nplp2-Gal4 line (w[1118] (I); P{w[+mC]=Nplp2-Gal4}vie72a (II); Korean Drosophila Resource Center Line #10023) to the UAS-Nplp2 RNAi line (y[1] v[1]; P{y[+t7.7] v[+t1.8]=TRiP.HMJ21484}attP40; Bloomington Stock Center Line #54041).

All behavioural experiments were performed on flies 3 days old.

Wild animals

This study did not use wild animals.

Reporting on sex

For all experiments, only males were used and analyzed, with the exception of detecting hydrocarbon abundance where females were collected and analyzed separately from males.

Field-collected samples

This study did not contain field-collected samples.

Ethics oversight

No ethical approval or guidance is required for Drosophila research at our institution at this time.

Note that full information on the approval of the study protocol must also be provided in the manuscript.
